# Impacts of community health education on the blood pressure levels and self-management abilities of elderly patients with hypertension

**DOI:** 10.3389/fmed.2026.1740150

**Published:** 2026-08-05

**Authors:** Shen Li

**Affiliations:** Tianshui Wulin Street Community Health Service Center, Hangzhou, Zhejiang, China

**Keywords:** blood pressure, community health education, elderly, hypertension, self-management

## Abstract

**Objective:**

The goal of this research was to explore the impacts of community health education on the blood pressure levels and self-management abilities of elderly patients suffering from hypertension.

**Methods:**

This study was a community-based, randomized controlled trial. Between January 2024 and January 2025, 200 hypertensive patients were randomly chosen from the Tianshui Wulin Street Community Health Service Center, to participate in the study. These patients were randomly assigned to either a control group (implemented routine nursing) or an intervention group (implemented community health education in addition to routine nursing). We compared the two groups in terms of blood pressure, self-care ability, self-management behavior, levels of depression and anxiety, medication adherence, sleep quality, quality of life, and satisfaction rates.

**Results:**

Six months post-intervention, both groups exhibited a decrease in systolic and diastolic blood pressure levels, PSQI scores and PHQ-9 and GAD-7 scores, as well as an increase in HBP SCP-behavior and HBP SCP-self-efficacy scores, HPSMBRS scores, TASHP scores, SF-36 scores (*p* < 0.05). Notably, compared with the control group, the intervention group demonstrated significantly lower systolic and diastolic blood pressure levels, higher HBP SCP-behavior and HBP SCP-self-efficacy scores, higher HPSMBRS scores, lower PHQ-9 and GAD-7 scores, higher TASHP scores, lower PSQI scores, higher SF-36 scores and a higher satisfaction rate 6 months post-intervention (*p* < 0.05).

**Conclusion:**

Community health education effectively lowers blood pressure levels, enhances self-care and self-management abilities, improves medication adherence, alleviates depression and anxiety, enhances sleep quality, boosts the quality of life, and increases satisfaction rates among elderly patients with hypertension.

## Introduction

Hypertension is a prevalent chronic condition, typically featured by a sustained elevation in blood pressure among affected individuals (1). Its main causes are genetic factors and unhealthy lifestyle habits, including diets high in salt, excessive alcohol intake, prolonged mental strain, as well as insufficient physical activity (2). Common symptoms of hypertension encompass headaches, fatigue or agitation, irregular heartbeat, palpitations, as well as tinnitus (3). Nevertheless, numerous individuals with hypertension may progress to developing serious complications without exhibiting any overt symptoms, such as strokes, blurred vision, loss of consciousness, and memory deficits. Consequently, hypertension has earned the moniker of the “silent killer” (4). The elderly population constitutes a high-risk group for hypertension (5). In 2010, it was estimated that globally, 1.39 billion adults were affected by hypertension, with a notably higher incidence in Low- and Middle-Income Countries (LMICs) (6). Over the past decades, China has also witnessed a rising prevalence of hypertension (7). Given its chronic nature, hypertension necessitates long-term management, with self-management practices playing a pivotal role in its control (8). According to the 2020 International Society of Hypertension global hypertension practice guidelines, in order to stabilize blood pressure, alleviate damage to target organs, as well as prevent other complications, all hypertensive patients should engage in varying degrees of self-management. This includes implementing a healthier lifestyle, keeping emotional equilibrium, regularly monitoring blood pressure, as well as adhering to standardized medication regimens (9). Nevertheless, elderly patients with hypertension often face challenges such as memory impairment, poor vision, diminished self-confidence, and may experience anxiety and depression. These factors can lead to non-adherence to prescribed medications and poor self-management, leading to inadequate blood pressure control and decreased quality of life (10). Consequently, it is of profound significance to assist this demographic in boosting their confidence, enhancing self-management skills, and improving medication compliance.

In this context, community health education, as an important part of hypertension management, has been receiving increasing attention (11). Through various forms such as organizing health lectures, distributing promotional materials, and providing individualized health guidance, community health education disseminates knowledge on hypertension prevention and treatment to elderly patients with hypertension, enhances their awareness of the disease, and strengthens their self-management ability (12). This not only helps improve the patients’ lifestyle, increase medication compliance, but also effectively promotes blood pressure control and promote the quality of life (13).

Nevertheless, at present, there exists a scarcity of comprehensive research examining the effects of community health education on both blood pressure levels and self-management capabilities among elderly hypertension patients in China. Consequently, our study was designed to delve into how community health education influences these aspects in this specific patient group, with the overarching goal of furnishing scientific evidence to refine hypertension management strategies.

## Methods

### Study design

This study was designed as a community-based, randomized controlled trial. Over the period from January 2024 to January 2025, we randomly recruited 200 hypertension patients from the Tianshui Wulin Street Community Health Service Center, to participate in our research. Inclusion criteria: (1) Patients diagnosed with primary hypertension; (2) Aged 65 years or older; (3) Currently taking at least one antihypertensive medication; (4) Possessing clear consciousness and stable health conditions; (5) Both patients and their family members have been informed and have consented to participate in the study. Exclusion criteria: (1) With secondary hypertension; (2) Those with severe impairments in liver or kidney function, or abnormal heart and lung functions; (3) Patients who are unable to care for themselves; (4) Individuals with mental illnesses, cognitive impairments, senile dementia, or malignant tumors; (5) Those with writing or verbal communication disabilities; (6) Elderly individuals without legal guardianship or those living alone.

### Sample size

Drawing upon the findings of a prior study concerning alterations in systolic blood pressure and diastolic blood pressure (14), we employed G*Power 3.1 software to compute the necessary sample size. We set the significance level (*α*) at 0.05 and the power (1 − *β*) at 0.8 (equivalent to *β* = 0.2), and took into account the expected dropout rate of 10%. As a result, this study required a total of 200 participants.

### Randomization

A scientifically rigorous random allocation method was adopted for grouping. Firstly, 200 hypertensive patients were numbered according to the order of enrollment, with the numbering range being 1–200. Then, using the random number generation function in statistical software (such as version 9.4 of SAS), 200 random numbers uniformly distributed between 0 and 1 were generated, and these random numbers were matched one-to-one with the patient numbers. Next, the patients were sorted according to the size of the generated random numbers. Patients with odd numbers in the sorted list were assigned to the control group, and those with even numbers were assigned to the intervention group, with 100 patients in each group.

### Methods

The control group implemented routine nursing intervention. Community medical staff conducted psychological intervention based on the disease situation of patients, informing patients of the correct medication methods, as well as the matters needing attention in daily life and diet. They also endeavored to gain the support and trust of the patients, analyzing their psychological problems and characteristics, and actively providing targeted guidance to alleviate the patients’ psychological stress and guide the release of negative emotions. At the same time, they distributed hypertension-related knowledge handbooks to patients and conducted publicity and education on hypertension-related knowledge.

Patients in the intervention group were provided with community health education in addition to routine nursing intervention.

1. Mental health education: Community medical staff actively communicated and interacted with patients, gained their trust, analyzed their psychological problems and characteristics, and provided targeted guidance to help them relieve negative emotions. At the same time, community medical staff organized common activities for patients to continuously enhance communication and interaction during the activities, thereby improving their mental health status. In addition, some patients are older and have poor memory, making it easy for them to forget medication. Therefore, regular reminders were necessary.
2. Blood pressure monitoring guidance: Community medical staff comprehensively understood the patient’s age, educational level, and comprehension ability, mastered the knowledge and precautions of blood pressure measurement, and guided the patient on how to use the blood pressure monitor correctly. If the patient needed to change the medication halfway, they needed to conduct blood pressure measurements at dawn, midday, evening, and before going to bed, respectively, for 2 weeks. If the blood pressure remained in a good state throughout this period, they measured it 3–5 times per week.
3. Medication education: The prevention and control of hypertension is a long-term process. Especially for patients with moderate or severe conditions, they need to adhere to lifelong medication intervention. Community medical staff focused on explaining the importance of continuous and regular medication use, detailing the usage, dosage, and precautions of the drugs for the patients, guiding them to master the methods of self-recognition of adverse drug reactions, strengthening publicity and education, stabilizing the patients’ blood pressure, and avoiding the occurrence of various complications. In case of patients stopping medication, community medical staff needed to first analyze the reasons, and then took targeted intervention measures based on the reasons, shortened the visit time, identified the problem and corrected it.
4. Exercise education: Community medical staff actively tailored exercise guidance to the specific conditions of patients with hypertension, such as age, physical condition, and exercise preferences. The specific exercise methods included walking slowly, table tennis, badminton, and Tai Chi. For patients of older age, it is recommended to mainly adopt the methods of slow walking and Tai Chi for exercise, 5 times per week, with the optimal duration being 30 min per session.
5. Dietary education: For patients with hypertension, controlling weight is of great significance. In the diet, the intake of salt should be controlled, and the intake of celery and bananas should be increased to supplement calcium and potassium. Additionally, community medical staff explained in detail to the patient the significance of a scientific diet, aiming to enhance the body’s immunity while ensuring adequate nutrition. To prevent patients from experiencing loss of appetite due to not consuming salt and limiting meat foods, it is necessary to actively maintain a diverse diet and strengthen the combination of foods. While achieving the control of unhealthy food intake, it is also necessary to ensure that patients receive sufficient nutrients.
6. Implementation of health education exchange activities: Community medical staff organized a patient exchange meeting every 2 months to popularize knowledge about hypertension, key points of prevention and treatment, high-risk factors, and the importance of following medical advice. Community medical staff provided health knowledge handbooks to patients and their families, and conducted follow-up visits via phone calls and home visits 1 month later to understand the patients’ mastery of disease knowledge, correct their bad behaviors, and supplement knowledge based on individual circumstances.

Both groups received an intervention for a period of 6 months.

### Observation indicators

1. We employed the AU-621 electronic sphygmomanometer (produced by A & D Medical Life source, Japan) to measure patients’ blood pressure in their right upper limbs. Prior to measurement, participants were instructed to rest for a minimum of 5 min. Two consecutive measurements were taken at least 30 s apart, with the average of these readings being recorded.
2. To evaluate self-care ability, we utilized the Chinese version of Hypertension Self-Care Profile (HBP SCP), which contains three scales: behavior, motivation, and self-efficacy. In our study, we focused on the Behavior and Self-efficacy subscales to measure self-care behavior and self-efficacy among hypertensive individuals, respectively. Each subscale has 20 items rated on a four-point scale, with higher scores indicating a greater level of self-care behavior or self-efficacy. The Cronbach’s alpha coefficients of the Chinese version of HBP SCP was 0.86 and 0.93 for the Behavior and Self-efficacy subscales, respectively (15).
3. We assessed self-management behavior using the Hypertension Patients Self-Management Behavior Rating Scale (HPSMBRS) developed by Zhao et al. (37). This scale, with an overall Cronbach’s *α* of 0.914 and validity of 0.910, encompasses six dimensions: disease monitoring, diet management, work and rest management, exercise management, emotion management, as well as medication management, totaling 33 items. The Likert 5-level scoring method was employed, with scores ranging from 1 (never) to 5 (always). The total scale score ranges from 33 to 165 points, with higher scores representing superior self-management behavior.
4. To measure perceived depressive and anxiety symptoms, we utilized the Patient Health Questionnaire-9 (PHQ-9) and the Generalised Anxiety Disorder Scale-7 (GAD-7). Items on both scales are rated on a four-point Likert scale, with higher scores indicating more severe symptoms of depression and anxiety, respectively. The Chinese version of PHQ-9 has a Cronbach’s alpha coefficient of 0.82 (17), while the GAD-7 has a Cronbach’s alpha coefficient of 0.90 (18).
5. The Therapeutic Adherence Scale for Hypertension Patients (TASHP), developed by Tang et al. (19), was employed to assess medication adherence. This scale comprises four dimensions: medication compliance, improper medication use, daily life management, and smoking and alcohol consumption management, totaling 25 items. Using the Likert 5-point scoring method, each item is assigned a score from 1 to 5. The total score ranges from 25 to 125. The overall scale has a Cronbach’s *α* of 0.86 and test–retest reliability of 0.96. Test–retest reliability was assessed by re-administering the scale to a subsample of 30 patients after a 2-week interval, yielding a coefficient of 0.96.
6. Sleep quality was evaluated using the Chinese version of the Pittsburgh Sleep Quality Index (PSQI), with Cronbach’s *α* coefficients ranging from 0.82 to 0.83 (20). The scale comprises 19 items, each scored from 0 to 3, with the total score ranging from 0 to 21. A higher PSQI global score indicates poorer subjective sleep quality.
7. To evaluate quality of life, we employed the Chinese version of the Short-Form 36 (SF-36) Health Survey, with Cronbach’s *α* coefficients above 0.7 (21). Each dimension is scored out of 100 points, with higher scores indicating better performance in that dimension.
8. The satisfaction rate was evaluated using a self-designed satisfaction assessment scale, with a total possible score of 100 points. A score of ≥80 indicates high satisfaction; a score between 60 and 79 indicates satisfaction; and a score <60 indicates dissatisfaction. The total satisfaction rate for nursing was calculated as (number of very satisfied + number of satisfied)/total number of cases × 100%.

### Statistical analysis

We employed SPSS 20.0 statistical software for data analysis. The normality of continuous variables was examined using the Shapiro–Wilk test. All quantitative variables in this study were found to follow a normal distribution (*p* > 0.05), therefore parametric tests were applied. For measurement data adhering to a normal distribution, results were presented as mean ± standard deviation (*x̄* ± *s*), and a *t*-test was utilized for comparing differences between groups. Count data were expressed in terms of the number of cases and corresponding percentages (%), with the *χ*^2^ test employed to assess intergroup differences. A *p*-value of less than 0.05 was considered indicative of statistically significant differences.

## Results

### Baseline data

No significant differences were seen in gender, age, course of disease, educational level and diabetes mellitus between the two groups (*p* > 0.05, Table 1), indicating the baseline data between the two groups were comparable.

**Table 1:** Baseline data between the two groups.

Items	Control group (*n* = 100)	Intervention group (*n* = 100)	*χ*^2^/*t*	*p*
Gender			0.183	0.668
Male	55 (55.00)	58 (58.00)		
Female	45 (45.00)	42 (42.00)		
Age (years)	68.45 ± 2.28	68.67 ± 2.32	0.676	0.499
Course of disease (years)	6.44 ± 0.13	6.48 ± 0.19	1.737	0.083
Educational level			0.181	0.913
Illiteracy	30 (30.00)	32 (32.00)		
Primary school	56 (56.00)	53 (53.00)		
Secondary or above	14 (14.00)	15 (15.00)		
Diabetes mellitus			0.202	0.653
Yes	32 (32.00)	35 (35.00)		
No	68 (68.00)	65 (65.00)		

### Blood pressure

**Figure 1:**
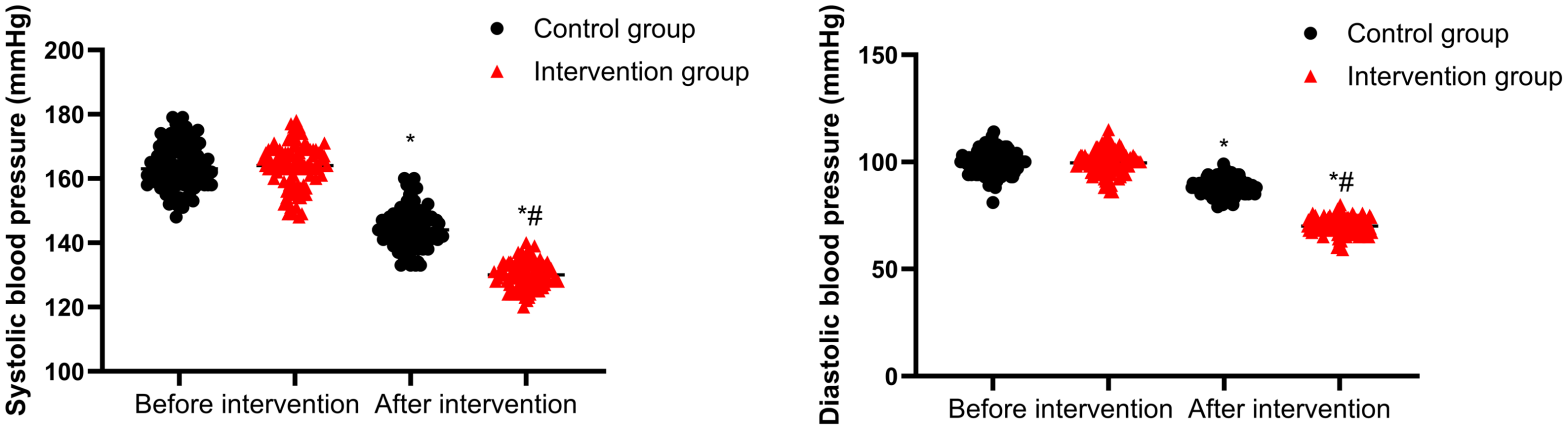
Blood pressure between the two groups before and 6 months after intervention. *p < 0.05, vs. before intervention; #*p* < 0.05, vs. control group.

At baseline, there were no significant differences in systolic or diastolic blood pressure between the two groups (*p* > 0.05). Six months following the intervention, both groups exhibited a reduction in blood pressure levels compared to their pre-intervention levels (*p* < 0.05). More importantly, the intervention group had lower blood pressure levels than the control group 6 months post-intervention (*p* < 0.05, Figure 1).

### Self-care ability

**Figure 2:**
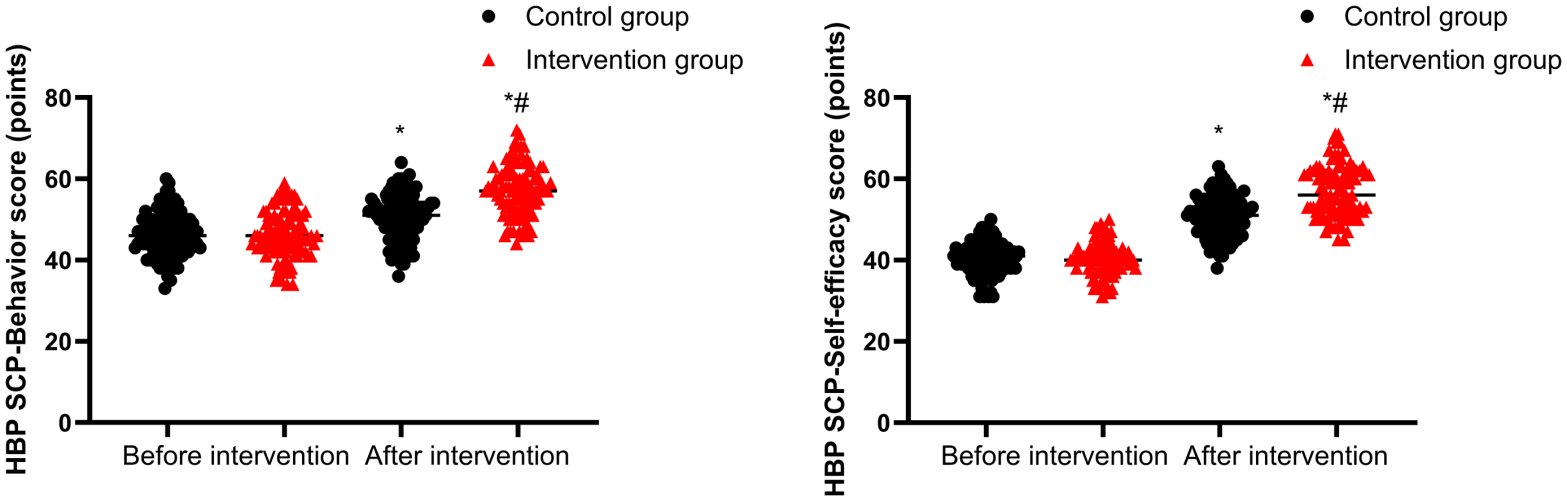
Self-care ability between the two groups before and 6 months after intervention. *p < 0.05, vs. before intervention; #*p* < 0.05, vs. control group.

At baseline, the HBP SCP-behavior and HBP SCP-self-efficacy scores did not differ significantly between the two groups (*p* > 0.05). After 6 months of intervention, both groups showed an increase in their HBP SCP-behavior and HBP SCP-self-efficacy scores compared to the pre-intervention levels (*p* < 0.05). Crucially, the intervention group achieved higher scores in both HBP SCP-behavior and HBP SCP-self-efficacy than the control group 6 months post-intervention (*p* < 0.05, Figure 2).

### Self-management behavior

**Figure 3:**
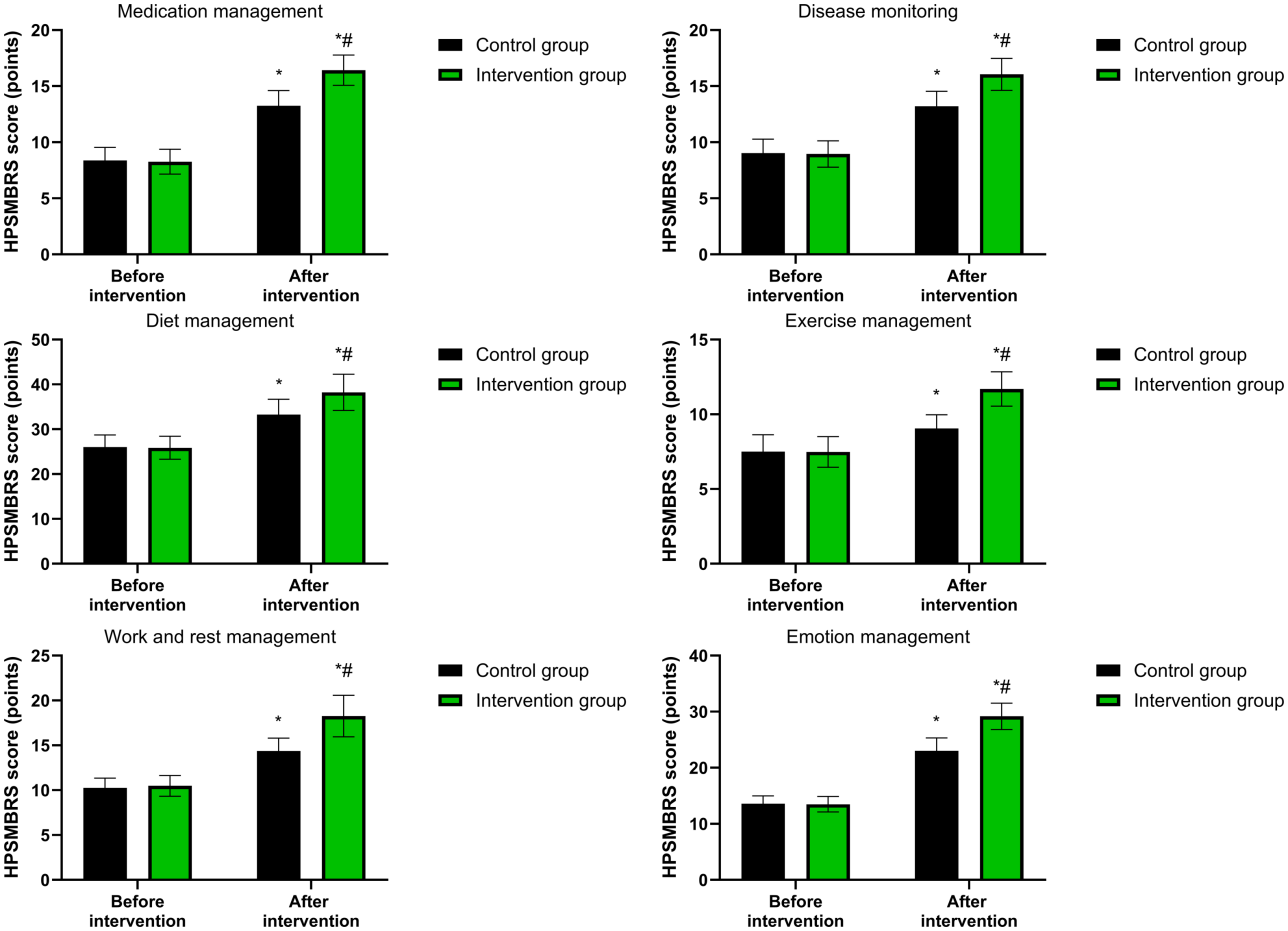
Self-management behavior between the two groups before and 6 months after intervention. *p < 0.05, vs. before intervention; #*p* < 0.05, vs. control group.

At baseline, no significant differences were found between the two groups in any dimension of the HPSMBRS or the total score (*p* > 0.05). Six months following the intervention, both groups demonstrated an increase in their HPSMBRS scores across all dimensions compared to their pre-intervention scores (*p* < 0.05). Notably, the intervention group achieved higher scores in each dimension of the HPSMBRS than the control group 6 months after the intervention (*p* < 0.05, Figure 3).

### Depression and anxiety

**Figure 4:**
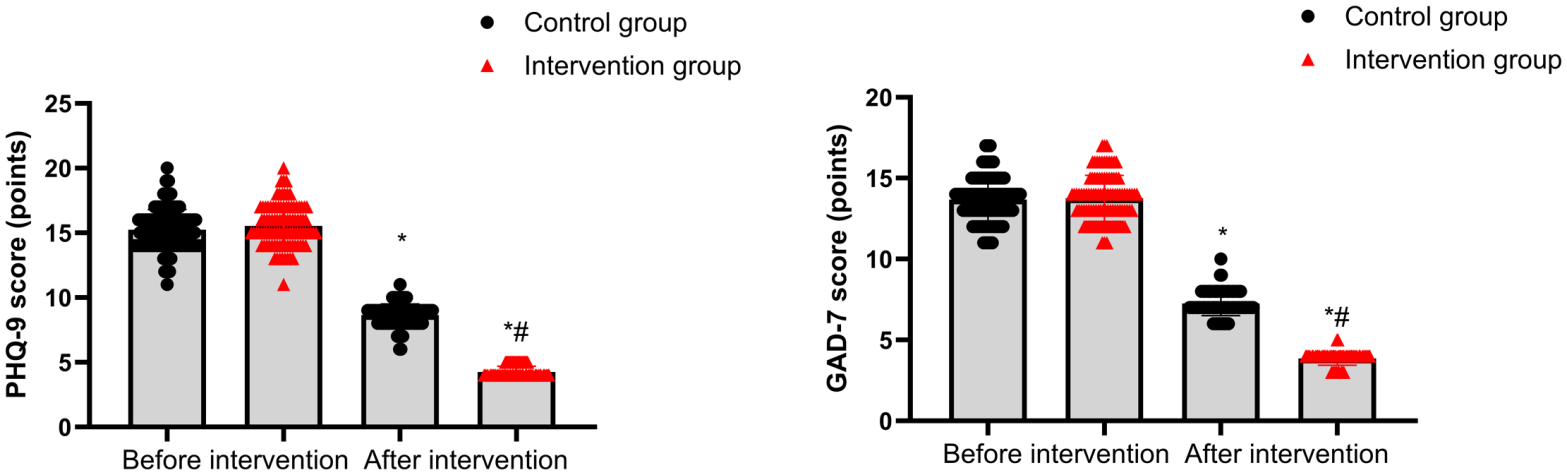
Depression and anxiety between the two groups before and 6 months after intervention. *p < 0.05, vs. before intervention; #*p* < 0.05, vs. control group.

At baseline, the PHQ-9 and GAD-7 scores were comparable between the two groups (*p* > 0.05). Six months post-intervention, both groups exhibited a reduction in their PHQ-9 and GAD-7 scores compared to their pre-intervention levels (*p* < 0.05). Critically, the intervention group had lower PHQ-9 and GAD-7 scores than the control group 6 months after the intervention (*p* < 0.05, Figure 4).

### Medication compliance

**Figure 5:**
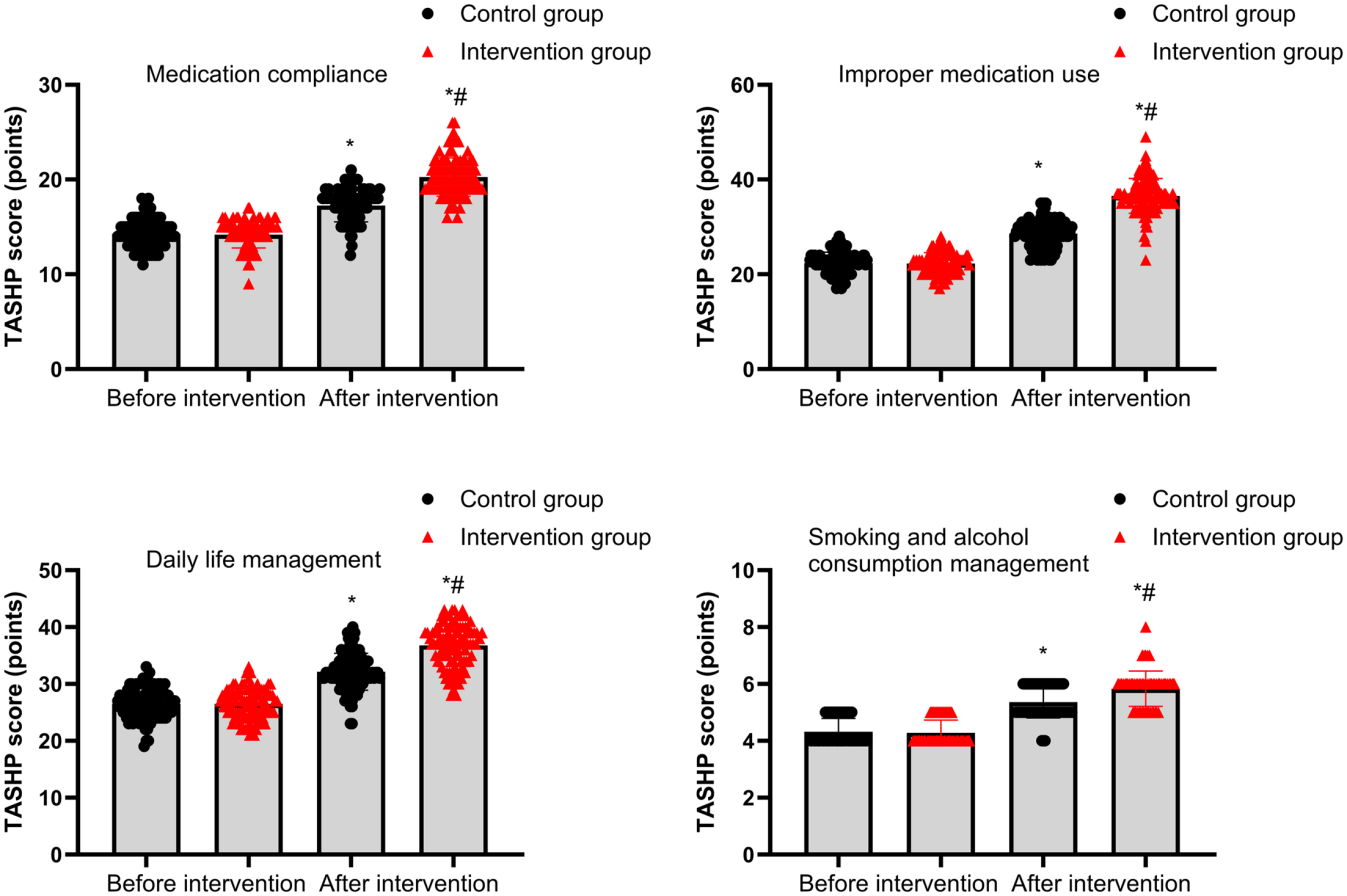
Medication compliance between the two groups before and 6 months after intervention. *p < 0.05, vs. before intervention; #*p* < 0.05, vs. control group.

At baseline, the TASHP scores in all dimensions did not differ significantly between the two groups (*p* > 0.05). After a 6-month intervention period, both groups showed a marked increase in their TASHP scores across all dimensions compared to their baseline scores (*p* < 0.05). Significantly, the intervention group achieved higher scores in each TASHP dimension than the control group 6 months following the intervention (*p* < 0.05, Figure 5).

### Sleep quality and quality of life

**Figure 6:**
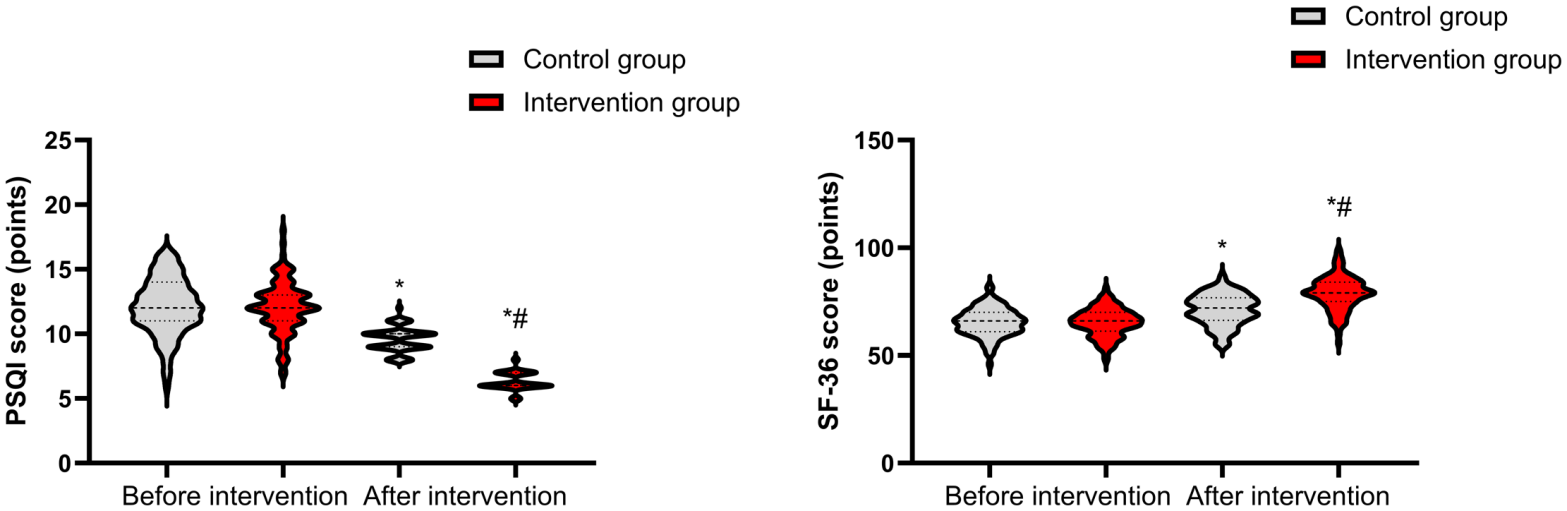
Sleep quality and quality of life between the two groups before and 6 months after intervention. *p < 0.05, vs. before intervention; #*p* < 0.05, vs. control group.

At baseline, there were no significant differences in PSQI and SF-36 scores between the two groups (*p* > 0.05). After 6 months of intervention, both groups exhibited a decrease in their PSQI scores compared to the pre-intervention levels (*p* < 0.05), while their SF-36 scores increased significantly from the baseline (*p* < 0.05). Notably, 6 months post-intervention, the intervention group demonstrated lower PSQI scores and higher SF-36 scores than the control group (*p* < 0.05, Figure 6).

### Patient satisfaction

The patient satisfaction was assessed at 6 months after the intervention. As indicated in Table 2, the satisfaction rate of the control group was 85.00% (85/100), and that of the intervention group was 96.00 (96/100). Compared with the control group, the intervention group had higher satisfaction rate (*p* < 0.05).

**Table 2:** Patient satisfaction between the two groups after the intervention.

Groups	Cases	Very satisfaction	Satisfaction	Dissatisfaction	Satisfaction rate
Control group	100	45 (45.00)	40 (40.00)	15 (15.00)	85 (85.00)
Intervention group	100	50 (50.00)	46 (46.00)	4 (4.00)	96 (96.00)
*χ* ^2^					7.036
*p*					0.008

## Discussion

Studies have indicated that the causes of hypertension in the elderly are complex, involving multiple factors such as age, genetics, lifestyle, and environment (22). As people age, the walls of blood vessels become hardened and lose their elasticity, increasing the risk of hypertension in the elderly (23). Additionally, poor lifestyle habits and environmental factors may also increase the likelihood of developing hypertension (24). After a diagnosis of hypertension, patients may experience negative emotions such as concerns about treatment efficacy, physical discomfort, resistance, or negativity, which may affect the treatment outcome (25). Therefore, implementing health education measures is crucial for improving this situation.

Health education, as an innovative nursing concept, has been widely adopted and implemented in clinical practice in recent years (26). It focuses on enhancing the health knowledge and behaviors of patients and their families during disease treatment, rehabilitation, and daily life (27). Unlike the conventional nursing model, health education places greater emphasis on patients’ self-management abilities, including emotional control and daily life management (28). The goal is to help patients develop a positive and optimistic attitude, rationally and scientifically understand their diseases, and work with the medical team to receive effective treatment (29). A large number of literature and clinical experiences have shown that health education, from multiple perspectives such as diet, exercise, and medication, enables patients to comprehensively understand the causes of diseases and appropriate lifestyles, effectively control the condition, as well as improve the quality of life (30). Moreover, health education not only promotes individual health but also contributes to improving the overall health level of the society (31). With the evolution of medical models and the enhancement of public health awareness, the role of health education in clinical nursing is becoming increasingly prominent (16).

In our study, the findings revealed 6 months post-intervention, both groups experienced a reduction in systolic and diastolic blood pressure compared to their pre-intervention levels. Crucially, the intervention group demonstrated lower systolic and diastolic blood pressure values than the control group 6 months post-intervention. These outcomes imply that community health education programs can effectively enhance blood pressure management among elderly patients with hypertension. In line with our findings, a cluster-randomized trial implemented by Khanal et al. demonstrated that health education, coupled with regular home visits by health volunteers, significantly improved hypertension awareness and reduced blood pressure levels among uncontrolled hypertensive patients at the community level in Nepal (14). Similarly, a systematic review by Israfil et al. highlighted the positive impact of mobile phone-based health education interventions in effectively controlling hypertension within community settings (32).

Our study revealed 6 months post-intervention, both groups exhibited higher scores in HBP SCP-behavior, HBP SCP-self-efficacy, HPSMBRS, and TASHP compared to their pre-intervention levels. Notably, the intervention group achieved higher scores in these areas than the control group 6 months post-intervention. These findings suggest that community health education programs can effectively enhance the self-care, self-management capabilities, as well as medication adherence of elderly patients with hypertension. Consistent with our results, Cui et al. indicated that a structured education program significantly improved medication adherence among rural patients with chronic heart failure (33). Similarly, Anggreini et al. found that health education interventions could enhance knowledge and self-care management practices related to hypertension in hypertensive patients (34).

Additionally, our study demonstrated 6 months post-intervention, both groups experienced a decline in PHQ-9, GAD-7, and PSQI scores compared to their pre-intervention levels, while SF-36 scores increased in both groups. Crucially, the intervention group exhibited significantly lower PHQ-9, GAD-7, and PSQI scores, along with higher SF-36 scores, than the control group 6 months post-intervention. Furthermore, the intervention group had a higher satisfaction rate than that of the control group. These findings highlight that community health education programs can effectively alleviate depression and anxiety, enhance sleep quality, improve overall quality of life, and boost satisfaction rates among elderly patients with hypertension. Similarly, Sun et al. reported that psychological nursing and health education interventions for elderly patients with lung cancer significantly improved negative moods, enhanced life quality, and contributed to a better prognosis (35). Additionally, Tao et al. found that empowerment theory-based health education improved treatment compliance and quality of life of elderly patients with type 2 diabetes mellitus (36).

In conclusion, community health education effectively lowers blood pressure levels, enhances self-care and self-management abilities, improves medication adherence, alleviates depression and anxiety, enhances sleep quality, boosts the quality of life, and increases satisfaction rates among elderly patients with hypertension.

## Data Availability

The datasets presented in this study can be found in online repositories. The names of the repository/repositories and accession number(s) can be found in the article/supplementary material.
